# Dementias show differential physiological responses to salient sounds

**DOI:** 10.3389/fnbeh.2015.00073

**Published:** 2015-03-24

**Authors:** Phillip D. Fletcher, Jennifer M. Nicholas, Timothy J. Shakespeare, Laura E. Downey, Hannah L. Golden, Jennifer L. Agustus, Camilla N. Clark, Catherine J. Mummery, Jonathan M. Schott, Sebastian J. Crutch, Jason D. Warren

**Affiliations:** ^1^Dementia Research Centre, UCL Institute of Neurology, University College LondonLondon, UK; ^2^London School of Hygiene and Tropical Medicine, University of LondonLondon, UK

**Keywords:** auditory looming, salience, nonverbal sound, pupillometry, dementia, semantic dementia, frontotemporal dementia, Alzheimer's disease

## Abstract

Abnormal responsiveness to salient sensory signals is often a prominent feature of dementia diseases, particularly the frontotemporal lobar degenerations, but has been little studied. Here we assessed processing of one important class of salient signals, looming sounds, in canonical dementia syndromes. We manipulated tones using intensity cues to create percepts of salient approaching (“looming”) or less salient withdrawing sounds. Pupil dilatation responses and behavioral rating responses to these stimuli were compared in patients fulfilling consensus criteria for dementia syndromes (semantic dementia, *n* = 10; behavioral variant frontotemporal dementia, *n* = 16, progressive nonfluent aphasia, *n* = 12; amnestic Alzheimer's disease, *n* = 10) and a cohort of 26 healthy age-matched individuals. Approaching sounds were rated as more salient than withdrawing sounds by healthy older individuals but this behavioral response to salience did not differentiate healthy individuals from patients with dementia syndromes. Pupil responses to approaching sounds were greater than responses to withdrawing sounds in healthy older individuals and in patients with semantic dementia: this differential pupil response was reduced in patients with progressive nonfluent aphasia and Alzheimer's disease relative both to the healthy control and semantic dementia groups, and did not correlate with nonverbal auditory semantic function. Autonomic responses to auditory salience are differentially affected by dementias and may constitute a novel biomarker of these diseases.

## Introduction

Accurate processing of salient sensory signals is essential in order to negotiate our physical and social environment successfully. Stimulus salience is carried by a variety of properties ranging from basic perceptual cues such as contrast (brightness, loudness) and motion (approach—withdrawal) to more complex semantic and affective attributes (Fletcher et al., in press); while the processing of salience engages distributed cortico-subcortical neural circuitry (Neuhoff, 2001; Seifritz et al., 2002; Bach et al., 2008; Seeley et al., 2009; Wang et al., 2012; Wang and Munoz, 2014). These circuits include cortical areas in the region of the temporo-parietal junction (in particular, the superior temporal sulcus) representing dynamic changes in auditory and visual stimuli and their crossmodal integration (Seifritz et al., 2002; Bach et al., 2008; Seeley et al., 2009; Tyll et al., 2013) and antero-mesial temporal lobe structures (notably, amygdala) and inferior frontal cortices mediating evaluation of the behavioral relevance of sensory signals (Seifritz et al., 2002; Bach et al., 2008; Fletcher et al., 2013; Tyll et al., 2013; Perry et al., 2014). Loss of awareness of salient signals or aberrant attribution of salience to banal stimuli due to involvement of the brain networks that code salience (Seifritz et al., 2002; Bach et al., 2008, 2009; Seeley et al., 2009; Kumfor and Piguet, 2012; Fletcher et al., 2013; Kumfor et al., 2013; Warren et al., 2013a,b; Perry et al., 2014; Zhou and Seeley, 2014) may contribute to the emotional, motivational and social deficits that often determine disability and burden in dementia diseases (Warren et al., 2013a). Such deficits remain poorly understood and difficult to measure, and there is accordingly considerable potential interest in resolving more basic pathophysiological mechanisms (such as abnormal salience coding) that might be used to generate indices of disease activity and candidate biomarkers.

Nonverbal sound is well fitted in evolutionary terms to generate salient sensory signals: sounds are our major source of information about the wider sensory environment under conditions of reduced vision, and whether a sound source is approaching or withdrawing is of fundamental behavioral relevance. Stimuli that approach or “loom” are more salient than those that recede: the ability to shift attention preferentially toward approaching stimuli carries a survival advantage, both for engaging with desirable stimuli and avoiding potential threats. The percept of looming can be generated robustly even from limited acoustic cues (for example, intensity ramps) suggesting that such simple cues may convey relevant salience information and evoke appropriate behavioral responses (Neuhoff, 1998; Seifritz et al., 2002; Bach et al., 2008). Preferential responsiveness to approaching vs. withdrawing sounds has been demonstrated across primate species: monkeys orientate longer toward increasing vs. decreasing intensity sounds (Schiff et al., 1962; Ghazanfar et al., 2002); while human subjects show lower thresholds for detection of intensity differences between static tones when intensity increases from the first to the second tone (Ellermeier, 1996) and rate dynamically approaching sounds as closer, louder, faster and more unpleasant, alerting and threatening than withdrawing sounds (Ellermeier, 1996; Neuhoff, 1998; Stecker and Hafter, 2000; Bach et al., 2008, 2009; Cappe et al., 2009). Besides evoking greater behavioral responses, looming sounds are more physiologically arousing, producing greater autonomic responses as indexed by changes in galvanic skin conductance and heart rate, than withdrawing sounds (Bach et al., 2008).

Abnormal cognitive and emotional responses to sounds are well documented in dementia diseases, particularly those in the frontotemporal lobar degeneration spectrum (Hoefer et al., 2008; Mahoney et al., 2011; Omar et al., 2011; Hsieh et al., 2012; Fletcher et al., 2013, in press). However, auditory salience coding and its physiological correlates have not been studied systematically in these diseases. It is now well established that neurodegenerative diseases target specific large-scale distributed brain networks (Seeley et al., 2009; Warren et al., 2013b; Zhou and Seeley, 2014) previously implicated in the processing of auditory salience in the healthy brain (Seifritz et al., 2002; Bach et al., 2008; Tyll et al., 2013). On both clinical and neuroanatomical grounds, dementia syndromes are therefore predicted to show separable profiles of abnormal auditory salience processing. To the extent that evaluation of the behavioral relevance of sounds is particularly critical, diseases that selectively target anterior temporal lobe circuits are predicted to show the most marked derangement of salience processing (Bach et al., 2008); whereas if the requirement to code dynamic acoustic cues is critical, diseases with heavier involvement of more posterior temporo-parietal and auditory association cortices would produce more marked deficits of auditory salience coding (Seifritz et al., 2002).

Here we addressed this issue in a cohort of patients representing canonical dementia syndromes in relation to healthy older individuals. We measured behavioral and autonomic (pupillometric) responses to looming vs. withdrawing sounds and compared these salience responses with an index of nonverbal auditory semantic function. We hypothesized that dementia diseases would show separable behavioral and physiological signatures of altered auditory salience coding. More specifically, we hypothesized that salience response profiles would differentiate dementia syndromes preferentially targeting more antero-mesial temporal and inferior frontal networks (semantic dementia, SD; behavioral variant frontotemporal dementia, bvFTD (Seeley et al., 2009; Fletcher and Warren, 2011; Warren et al., 2013b; Zhou and Seeley, 2014) from syndromes targeting more posterior and dorsal brain networks (progressive nonfluent aphasia, PNFA; Alzheimer's disease, AD (Grossman, 2012; Warren et al., 2012, 2013b) previously implicated in different aspects of the analysis of looming sounds (Seifritz et al., 2002; Bach et al., 2008; Tyll et al., 2013). As auditory salience processing has a modular organization, we further predicted that autonomic correlates of auditory salience should be at least in part dissociable from behavioral and cognitive indices of nonverbal sound analysis.

## Methods

### Participant details

Consecutive patients fulfilling consensus diagnostic criteria (Dubois et al., 2007; Gorno-Tempini et al., 2011; Rascovsky et al., 2011) for SD (*n* = 10), probable bvFTD (*n* = 16), PNFA (*n* = 12) or typical amnestic AD (*n* = 10) and 26 healthy older individuals with no history of neurological or psychiatric illness participated. Genetic screening of the cohort revealed 12 patients with pathogenic mutations, all in the bvFTD group (six with expansions in the C9orf72 gene, six with mutations in the MAPT gene). Cerebrospinal fluid amyloid-beta_1−42_ and tau ratios were available for six patients with AD, seven with bvFTD, seven with PNFA and three with SD, and were in keeping with the clinical diagnosis in all cases. No participant had a clinical history of hearing abnormalities; in order to assess any effect from peripheral hearing function on experimental performance, screening pure tone audiometry was conducted in each group using a previously described procedure (Goll et al., 2010). Nine patients with AD, six with bvFTD, two with PNFA and one with SD were receiving treatment with acetylcholinesterase inhibitors. All participants had a comprehensive assessment of general neuropsychological functions (Table 1). Syndromic diagnoses were further substantiated by structural volumetric brain MRI which showed compatible profiles of regional atrophy in all cases with minimal or mild coexisting cerebrovascular damage.

**Table 1:** **Demographic, clinical and general neuropsychological data for participant groups: Maximum scores on neuropsychological tests are shown in parentheses; mean (standard deviation) data are shown unless otherwise indicated**.

**Characteristic**	**Controls**	**SD**	**bvFTD**	**PNFA**	**AD**
**GENERAL**					
No.	26	10^*^	16	12^**^	10^*^
Gender distribution (f:m)	12:14	6:4	**3:11**	**3:9**	5:5
Age (yrs): mean (range)	67 (57 –74)	65 (56 –78)	66 (52 – 84)	68 (57–79)	66 (60–78)
Education (yrs)	16.6 (2.0)	15.0 (3.2)	14.6 (3.4)	15 (3.1)	15.3 (2.4)
Symptom duration (yrs)	NA	4.5 (2.1)	8.3 (6.2)	4.3 (2.1)	5.3 (2.1)
MMSE (range)	29.7 (29–30)	**20.2 (18–30)**	**24.1 (9–27)**	**25.4 (14–29)**	**23.8 (21–29)**
**IQ**					
Verbal	123 (8.2)	**81 (17)**	**89 (20)**	**77 (15)**	**101 (14)**
Performance	119 (14)	**111 (16)**	**97 (17)**	**98 (17)**	**90 (16)**
**EPISODIC MEMORY**					
RMT words (/50)	47 (3)	**30 (8)**	**35 (6)**	40 (8)	**30 (5)**
RMT faces (/50)	44 (4)	**36 (8)**	**34 (6)**	**38 (5)**	**32 (5)**
**SEMANTIC PROCESSING**					
BPVS (/150)	148 (2)	**103 (45)**	**132 (15)**	**132 (24)**	**140 (8)**
Sound classification task^†^ (45)	89 (5)	**71 (10)**	**78 (12)**	**82 (7)**	83 (6)
**EXECUTIVE FUNCTION**					
D-KEFS Stroop word	21 (4)	26 (9)	**27 (9)**	**50 (14)**	**31 (9)**
D-KEFS Stroop inhibition	57 (16)	77 (34)	**94 (42)**	**118 (51)**	**116 (47)**
Digit span reverse (max)	5 (1)	5 (2)	5 (1)	**3 (1)**	5 (2)
**VISUOSPATIAL**					
VOSP (/20)	18 (2)	16 (3)	17 (2)	16 (2)	**16 (2)**

*Significant group differences in patients relative to healthy controls are in bold*.

* general neuropsychological data in 9 patients

** general neuropsychological data in 10 patients;

† *experimental nonverbal auditory semantic task (see text); AD, amnestic Alzheimer's disease; BPVS, British Picture Vocabulary Scale; bvFTD, behavioral variant frontotemporal dementia; D-KEFS, Dellis-Kaplan Executive Function System; MMSE, Mini-Mental State Examination score; NA, not applicable; PNFA, progressive nonfluent aphasia; RMT, Recognition Memory Test; SD, semantic dementia; VOSP, Visual Object and Spatial Perception battery*.

Written informed consent was obtained for all participants in accordance with the Declaration of Helsinki; the study was approved by the UCL/UCLH Joint Research Ethics Committee.

### Assessment of nonverbal auditory semantic function

In order to assess nonverbal auditory semantic competence when interpreting behavioral and pupillometric correlates of auditory salience processing, we created a novel, within-modality, semantic matching (classification) task on highly identifiable, real nonverbal sounds (accordingly, this stimulus set did not include the synthetic tones used to assess salience responses in the pupillometry experiment; see below). Sixty serial sound pairs (see Supplementary Table S1) were presented in randomized order; the task on each trial was to classify the paired sounds according to whether they were associated with the same sound source or with different sources (“Are the sounds made by the same kind of thing or by different kinds of things?”). No feedback about performance was given and no time limits on responses were imposed.

### Pupillometry experiment

To assess auditory salience processing, we synthesized digital sounds under two conditions: “approaching” or “looming” (tones with increasing intensity) vs. “withdrawing” (tones with decreasing intensity). Carrier sound stimuli were synthesized as pure tone wavefiles under Matlab 7.0^®^ (http://www.mathworks.co.uk/) at base frequency 700 or 1000 Hz; narrow-band sounds in this frequency range have been shown previously to evoke robust behavioral and physiological responses to auditory looming in the healthy brain (Neuhoff, 1998; Seifritz et al., 2002; Bach et al., 2008, 2009). All tones were 2 s in duration with the same base mean (root-mean-square) intensity level. Intensity changes were applied as linear ramps between 0 and 75 decibels with 5 ms onset and offset ramps to eliminate click artifacts. These large intensity changes were easily perceived by all participants: sounds with increasing or decreasing intensity were perceived as “approaching” or “withdrawing,” respectively.

Six synthetic sound stimuli (three tones representing each of the two salience conditions) were presented in randomized order interspersed with a playlist of 30 familiar nonverbal sounds (representing common human vocal, animal, mechanical and environmental noises, included in order to improve estimation of stable baseline pupillometry responses while minimizing effects from habituation); successive synthetic sound stimuli were separated by at least three intervening natural sounds.

During pupillometry, the participant was seated approximately 1 m from a large desktop computer screen in a dimly and uniformly illuminated room. Pupil area was measured from the right pupil using an infra-red camera [Eyelink II; SR Research, Canada] mounted on a headset just below the line of sight while the participant fixated a white circle (diameter 1 cm) in the center of the monitor. Each experimental trial was triggered once adequate visual fixation was achieved and pupil area was measured (sampling rate 250 Hz) over the entire trial duration using Eyelink II software. During each trial there was an initial brief silent interval (2 s), followed by the sound stimulus (2 s) and a final silent equilibration interval (7 s). Synthetic sound stimuli were presented via headphones [Audio-Technica ATH-M50] from a notebook computer at a constant, comfortable baseline listening level (at least 70 decibels). On completion of each trial, a modified Likert pictorial scale was displayed and the participant rated how alerting they found the stimulus using a wireless mouse; a participant response signaled the next trial. All pupillometry and behavioral data were recorded for off-line analysis.

### Pre-processing of pupillometry data

A customized algorithm written in STATAv12.1^®^ was used to calculate maximal pupil change (dilatation) from baseline area for each trial; baseline values were calculated as the mean value over the initial 2 s silent interval of the trial. Artifacts were chiefly blinks, easily detected due to their characteristic rapid time course; pupil data were discarded over the interval 50 ms prior to 750 ms following the artifact, to allow for completion of an ensuing light reflex (as determined from data collected in a healthy control pilot group). The total proportions of data points removed due to artifacts did not differ significantly between sounds or between experimental groups. There was a strong correlation between baseline and maximal pupil area over the course of the experiment and across participants; accordingly, a log transform was used to generate a metric of pupil response.

### Data analyses

Demographic characteristics, general neuropsychological and nonverbal auditory semantic performance, medication use and peripheral hearing function were compared between participant groups and correlations with pupil responses were assessed over the combined patient cohort using linear regression models. Behavioral rating and pupil responses in approaching and withdrawing sound conditions were compared for individual participants within each group using linear fixed effects models with crossed fixed effects for participant and item (after exclusion of interleaved natural sounds), in order to account for non-independence of within-participant correlations of behavioral ratings and pupil responses. For each individual, a measure of the magnitude of the difference in condition responses was calculated by subtracting mean response to withdrawing sounds from mean response to approaching sounds; these individual difference measures were entered into the group-wise analysis, and groups were compared using linear regression (effectively implementing group-by-condition interactions). Sound position within the experimental playlist was incorporated as a nuisance covariate in sound condition comparisons; gender was incorporated as a nuisance covariate in group comparisons, given previous evidence that females and males perceive looming sounds differently (Neuhoff et al., 2009). For all analyses, *p* < 0.05 was adopted as the threshold for reporting statistically significant effects.

## Results

### General characteristics of participant groups

Demographic, clinical and general neuropsychological data are summarized in Table 1. Participant groups did not differ in mean age but did differ in gender distribution [males were relatively over-represented in the bvFTD group relative both to the healthy control group (*p* = 0.02) and the PNFA group (*p* = 0.03)]. Patient groups did not differ in mean symptom duration. Clinical syndromic diagnoses were corroborated by findings on standard general neuropsychological assessment.

Baseline peripheral hearing thresholds did not vary between patient groups. The SD, bvFTD and PNFA groups showed impaired semantic classification of nonverbal sounds relative to the healthy older control group (*p* < 0.0001; see Table 1); no nonverbal auditory semantic deficit was demonstrated in the AD group.

### Behavioral ratings

**Figure 1:**
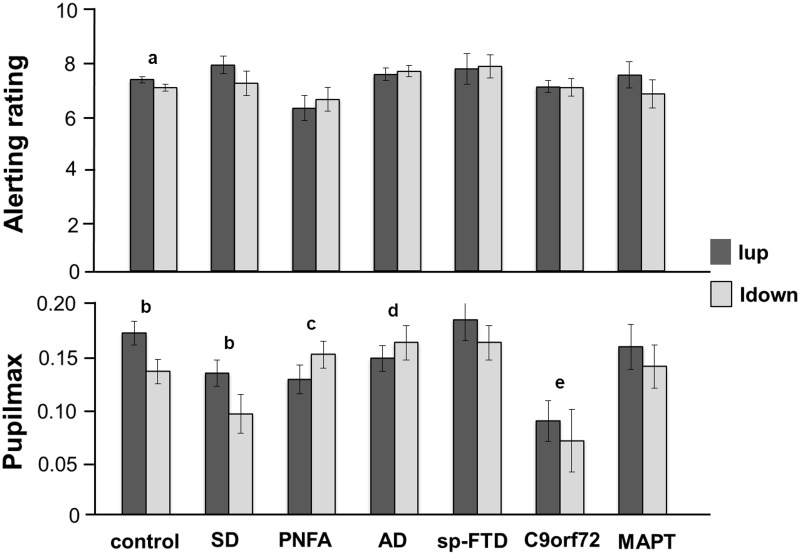
**Mean alerting ratings (upper panel) and maximal pupil responses (lower panel) for the experimental groups for approaching (intensity increasing, Iup, dark gray) and withdrawing (intensity decreasing, Idown, light gray) sound conditions**. Alerting ratings are on a Likert scale (1, not all alerting; 10, highly alerting) and pupil responses are shown as log percentage maximal area change from baseline (Pupilmax). Mean values are shown (error bars signify 1 standard error). Key: a, strong trend (*p* < 0.07) to greater alerting ratings for Iup than Idown sounds; b, significantly greater (*p* < 0.05) pupil responses to Iup than Idown sounds; c, differential response to Iup vs. Idown sounds significantly reduced (*p* < 0.05) relative to healthy control and SD groups; d, differential response to Iup vs. Idown sounds significantly reduced (*p* < 0.05) relative to healthy control group; e, overall pupil responses significantly reduced (*p* < 0.05) relative to healthy older controls and sporadic bvFTD subgroup; AD, Alzheimer's disease; control, healthy older control group; C9orf72, behavioral variant frontotemporal dementia with C9orf72 gene mutations; MAPT, behavioral variant frontotemporal dementia with MAPT gene mutations; PNFA, progressive nonfluent aphasia; SD, semantic dementia; sp-FTD, sporadic behavioral variant frontotemporal dementia (no identified genetic mutation).

Behavioral alerting rating and pupil response data are summarized in Figure 1 and tabulated in Supplementary Table S2.

The PNFA group rated approaching sounds as significantly less alerting (*p* = 0.04) than did the healthy control group and showed a strong trend (*p* = 0.055) to rate approaching sounds as significantly less alerting than the SD group. There were no other syndromic group differences in mean overall alerting ratings for either sound condition. The healthy control group showed a strong trend to rate approaching sounds as significantly more alerting than withdrawing sounds (*p* = 0.07). No patient group showed a significant mean difference in alerting ratings between the two sound conditions; the magnitude of this behavioral condition difference did not differ between syndromic groups.

### Pupil responses

Baseline pupil size did not change systematically over the course of the experiment. There was no relationship between peripheral hearing thresholds or use of acetylcholinesterase inhibitors and baseline pupil size or condition-associated pupil responses. Comparing pupil responses between groups, overall pupil reactivity (i.e., mean pupil response to sounds irrespective of sound condition) did not differ between syndromic groups. The bvFTD subgroup with C9orf72 mutations had significantly reduced overall pupil responses relative both to healthy controls (*p* = 0.01) and to patients with sporadic bvFTD (*p* = 0.02) but no significant difference relative to other patient groups.

In the healthy control group, approaching sounds evoked significantly greater pupil dilatation (*p* < 0.01) than withdrawing sounds. The SD group (but no other syndromic group or genetic subgroup) retained the normal profile of significantly greater pupil responses to approaching than withdrawing sounds (*p* = 0.02). Relative to healthy controls, the magnitude of this differential pupil response to approaching vs. withdrawing sounds was significantly reduced in the PNFA group (*p* < 0.01) and in the AD group (*p* = 0.02). Relative to the SD group, the pupil condition difference was significantly reduced in the PNFA group (*p* = 0.02) and showed a strong trend to be significantly reduced in the AD group (*p* = 0.06). There were no other significant differences in pupil responses between groups.

There were no significant correlations between pupil response and disease duration, overall disease severity (as indexed by Mini-Mental State Examination score), general semantic (British Picture Vocabulary Scale) or nonverbal auditory semantic (sound classification test) performance over the patient cohort.

## Discussion

Here we have demonstrated that dementia syndromes have dissociable profiles of behavioral and autonomic reactivity to salient sounds. Whereas healthy older individuals (in line with previous work in younger cohorts: (Ellermeier, 1996; Neuhoff, 1998; Stecker and Hafter, 2000; Bach et al., 2008, 2009; Cappe et al., 2009) showed an enhanced behavioral response to approaching vs. withdrawing sounds, this differential behavioral response was lost across dementia syndromes. However, patients with SD (like healthy individuals) showed greater pupil dilatation in response to looming sounds than withdrawing sounds, whereas this salience signal was reduced (relative both to healthy controls and patients with SD) in PNFA and AD. Moreover, the physiological salience response in SD and pupil responses in the wider patient cohort did not correlate with nonverbal auditory semantic function. Taken together, these findings support a functional fractionation of human brain systems for the behavioral evaluation and physiological coding of sounds: physiological markers (in SD) may continue to signal the salience of sensory stimuli even where explicit evaluation is disrupted. The findings corroborate previous evidence for altered behavioral and physiological coding of emotional sounds in dementias (Fletcher et al., in press) and further suggest that physiological signatures based, for example, on autonomic salience responses may stratify dementia syndromes, with potential utility as biomarkers.

The finding of preserved auditory salience responses in SD and reduced responses in PNFA and AD corroborates our prior hypothesis concerning dissociable syndromic profiles of salience coding in these dementia syndromes. This syndromic pattern is intriguing in light of previous neuroanatomical work in the healthy brain (Bach et al., 2008). Anterior and mesial temporal structures (such as amygdala) that evaluate the behavioral and emotional salience of sensory stimuli (Bach et al., 2008) are typically heavily involved by the pathological process in SD [9] (and were involved on MRI in all cases here) but less consistently damaged in other dementia syndromes; in contrast, posterior temporo-parietal and fronto-parietal circuitry implicated in spatial analysis, orienting and evaluative behaviors (Downar et al., 2000; Seifritz et al., 2002; Bach et al., 2008) are typically involved in AD and PNFA but relatively spared in SD (Grossman, 2012; Warren et al., 2012, 2013a). Though any anatomical conclusion here must be tentative, our findings suggest that more posterior cortices (perhaps auditory association areas) (Kucyi et al., 2012) may play a key role in coding physiological responses to salient sounds but leave open the critical locus for generating evaluative behavioral responses (which were impaired across dementia syndromes here).

From a clinical perspective, our findings suggest that physiological signatures may help to define dementia diseases both syndromically and by molecular substrates. Molecular substrates are most clearly established for genetic mutation subgroups, for SD (clinically typical cases are generally underpinned by TDP-43 type C pathology: (Rohrer et al., 2011) and for clinically typical AD, when supported (as here) by cerebrospinal fluid parameters. These diseases were distinguished in the present study by profiles of overall pupil reactivity to sound (reduced in patients with C9orf72 mutations relative to healthy older adults and patients with sporadic bvFTD) and pupil salience responses (reduced in PNFA and AD relative to SD). It is noteworthy that pupil responses suggested some stratification of genetic subgroups within the pathologically and anatomically diverse bvFTD syndromic group. Such molecular stratification could help to resolve certain apparent inconsistencies with respect to autonomic reactivity profiles attributed to combined bvFTD syndromic cohorts in previous work: baseline autonomic (skin conductance) reactivity has been reported to be decreased in bvFTD (Robles et al., 1999; Joshi et al., 2014; Struhal et al., 2014), while autonomic responses to salient sounds have been reported to be depressed (Hoefer et al., 2008) or retained (Sturm et al., 2006). Physiological signatures may be particularly pertinent where diseases overlap clinically and anatomically (for example, sporadic and C9orf72-associated bvFTD) or where differentiation of molecular pathologies is currently difficult due to convergent phenotypic effects, particularly in bvFTD (Warren et al., 2013a,b). Physiological biomarkers could potentially have a role both in disease detection and in tracking syndromes or evaluating therapies, for example in the context of clinical trials targeted to particular proteinopathies. This could apply in more advanced disease, where conventional cognitive and structural neuroanatomical metrics lack sensitivity and specificity. More generally, however, there is a need for new, functionally relevant biomarkers that can reflect the impact of therapies dynamically, while potential for reversal of disease effects is maximal. In addition, the present findings may have future implications for symptomatic interventions based on manipulation of sensory salience (for example, warning signals).

The present study has several limitations that suggest directions for future work. Patient numbers here were small; with particular regard to the genetic subgroups here, the findings should be regarded as preliminary and await substantiation in larger patient cohorts. In the case of less common dementias, pooling of cohorts via multi-center collaborations (Rohrer et al., 2015) is likely to be required in order to power studies adequately to detect weaker disease effects. Moreover, our patients were studied within a relatively limited window of clinically established disease and without direct neuroanatomical or pathological substantiation. We adopted a single model physiological paradigm: pupillometry of salient sounds. Further work should evaluate physiological response profiles longitudinally over the course of disease (including presymptomatic, genetically at-risk cases), with other physiological metrics and in different sensory modalities, and with neuroanatomical and (ultimately) histopathological correlation.

## Author contributions

All authors fulfill the criteria of authorship and no-one else who fulfills the criteria has been excluded. PF, SC, and JW had the idea for the study and jointly designed the experiments. PF, CM, JS, and JW conducted clinical assessments. HG, LD, JA, and SC were involved in collecting and analysing behavioral data. Timothy Shakespeare assisted with pupillometry experiments and JN provided statstitical advice and assisted with data analysis. All authors were involved in writing and critically revising the article, and all have approved the final submitted version. JW accepts full responsibility for the work and controlled the decision to publish.

### Conflict of interest statement

All authors declare financial support for the submitted work from Wellcome Trust and the Medical Research Council. JW has received research grants from the Wellcome Trust, the Medical Research Council and Alzheimer's Research UK. SC has received grants from Alzheimer's research UK.

## References

[B1] Bach D. R. Neuhoff J. G. Perrig W. Seifritz E. (2009). Looming sounds as warning signals: the function of motion cues. Int. J. Psychophysiol. 74, 28–33. 10.1016/j.ijpsycho.2009.06.004 19615414

[B2] Bach D. R. Schachinger H. Neuhoff J. G. Esposito F. Di Salle F. Lehmann C.. (2008). Rising sound intensity: an intrinsic warning cue activating the amygdala. Cereb. Cortex 18, 145–150. 10.1093/cercor/bhm040 17490992

[B3] Cappe C. Thut G. Romei V. Murray M. M. (2009). Selective integration of auditory-visual looming cues by humans. Neuropsychologia 47, 1045–1052. 10.1016/j.neuropsychologia.2008.11.003 19041883

[B4] Downar J. Crawley A. P. Mikulis D. J. Davis K. D. (2000). A multimodal cortical network for the detection of changes in the sensory environment. Nat. Neurosci. 3, 277–283. 10.1038/72991 10700261

[B5] Dubois B. Feldman H. H. Jacova C. Dekosky S. T. Barberger-Gateau P. Cummings J.. (2007). Research criteria for the diagnosis of Alzheimer's disease: revising the NINCDS-ADRDA criteria. Lancet Neurol. 6, 734–746. 10.1016/S1474-4422(07)70178-3 17616482

[B6] Ellermeier W. (1996). Detectability of increments and decrements in spectral profiles. J. Acoust. Soc. Am. 99, 3119–3125 10.1121/1.414797

[B7] Fletcher P. D. Downey L. E. Witoonpanich P. Warren J. D. (2013). The brain basis of musicophilia: evidence from frontotemporal lobar degeneration. Front. Psychol. 4:347. 10.3389/fpsyg.2013.00347 23801975 PMC3689257

[B8] Fletcher P. D. Nicholas J. M. Shakespeare T. J. Downey L. E. Golden H. L. Agustus C. N. (in press). Physiological phenotyping of dementias using emotional sounds. Alz. Dem. Diag. Assess. Dis. Mon. 10.1016/j.dadm.2015.02.003 PMC4629103 26634223

[B9] Fletcher P. D. Warren J. D. (2011). Semantic dementia: a specific network-opathy. J. Mol. Neurosci. 45, 629–636. 10.1007/s12031-011-9586-3 21710360 PMC3207124

[B10] Ghazanfar A. A. Neuhoff J. G. Logothetis N. K. (2002). Auditory looming perception in rhesus monkeys. Proc. Natl. Acad. Sci. U.S.A. 99, 15755–15757. 10.1073/pnas.242469699 12429855 PMC137788

[B11] Goll J. C. Crutch S. J. Loo J. H. Rohrer J. D. Frost C. Bamiou D. E.. (2010). Non-verbal sound processing in the primary progressive aphasias. Brain 133(Pt 1), 272–285. 10.1093/brain/awp235 19797352 PMC2801322

[B12] Gorno-Tempini M. L. Hillis A. E. Weintraub S. Kertesz A. Mendez M. Cappa S. F.. (2011). Classification of primary progressive aphasia and its variants. Neurology 76, 1006–1014. 10.1212/WNL.0b013e31821103e6 21325651 PMC3059138

[B13] Grossman M. (2012). The non-fluent/agrammatic variant of primary progressive aphasia. Lancet Neurol. 11, 545–555. 10.1016/S1474-4422(12)70099-6 22608668 PMC3361730

[B14] Hoefer M. Allison S. C. Schauer G. F. Neuhaus J. M. Hall J. Dang J. N.. (2008). Fear conditioning in frontotemporal lobar degeneration and Alzheimer's disease. Brain 131(Pt 6), 1646–1657. 10.1093/brain/awn082 18492729 PMC2544622

[B15] Hsieh S. Foxe D. Leslie F. Savage S. Piguet O. Hodges J. R. (2012). Grief and joy: emotion word comprehension in the dementias. Neuropsychology 26, 624–630. 10.1037/a0029326 22823134

[B16] Joshi A. Mendez M. F. Kaiser N. Jimenez E. Mather M. Shapira J. S. (2014). Skin conductance levels may reflect emotional blunting in behavioral variant frontotemporal dementia. J. Neuropsychiatry Clin. Neurosci. 26, 227–232. 10.1176/appi.neuropsych.12110332 25093763

[B17] Kucyi A. Hodaie M. Davis K. D. (2012). Lateralization in intrinsic functional connectivity of the temporoparietal junction with salience- and attention-related brain networks. J. Neurophysiol. 108, 3382–3392. 10.1152/jn.00674.2012 23019004

[B18] Kumfor F. Irish M. Hodges J. R. Piguet O. (2013). The orbitofrontal cortex is involved in emotional enhancement of memory: evidence from the dementias. Brain 136, 2992–3003. 10.1093/brain/awt185 23838694

[B19] Kumfor F. Piguet O. (2012). Disturbance of emotion processing in frontotemporal dementia: a synthesis of cognitive and neuroimaging findings. Neuropsychol. Rev. 22, 280–297. 10.1007/s11065-012-9201-6 22577002

[B20] Mahoney C. J. Rohrer J. D. Goll J. C. Fox N. C. Rossor M. N. Warren J. D. (2011). Structural neuroanatomy of tinnitus and hyperacusis in semantic dementia. J. Neurol. Neurosurg. Psychiatry 82, 1274–1278. 10.1136/jnnp.2010.235473 21531705 PMC3188784

[B21] Neuhoff J. G. (1998). Perceptual bias for rising tones. Nature 395, 123–124. 10.1038/25862 9744266

[B22] Neuhoff J. G. (2001). An adaptive bias in the perception of looming auditory motion. Ecol. Psychol. 13, 87–110 10.1207/S15326969ECO1302_2

[B23] Neuhoff J. G. Planisek R. Seifritz E. (2009). Adaptive sex differences in auditory motion perception: looming sounds are special. J. Exp. Psychol. Hum. Percept. Perform. 35, 225–234. 10.1037/a0013159 19170484

[B24] Omar R. Henley S. M. Bartlett J. W. Hailstone J. C. Gordon E. Sauter D. A.. (2011). The structural neuroanatomy of music emotion recognition: evidence from frontotemporal lobar degeneration. Neuroimage 56, 1814–1821. 10.1016/j.neuroimage.2011.03.002 21385617 PMC3092986

[B25] Perry D. C. Sturm V. E. Seeley W. W. Miller B. L. Kramer J. H. Rosen H. J. (2014). Anatomical correlates of reward-seeking behaviours in behavioural variant frontotemporal dementia. Brain 137(Pt 6), 1621–1626. 10.1093/brain/awu075 24740987 PMC4032100

[B26] Rascovsky K. Hodges J. R. Knopman D. Mendez M. F. Kramer J. H. Neuhaus J.. (2011). Sensitivity of revised diagnostic criteria for the behavioural variant of frontotemporal dementia. Brain 134(Pt 9), 2456–2477. 10.1093/brain/awr179 21810890 PMC3170532

[B27] Robles A. Tourino R. Gude F. Noya M. (1999). The tropicamide test in patients with dementia of Alzheimer type and frontotemporal dementia. Funct. Neurol. 14, 203–207. 10713893

[B28] Rohrer J. D. Nicholas J. M. Cash D. M. van Swieten J. Dopper E. Jiskoot L.. (2015). Presymptomatic cognitive and neuroanatomical changes in genetic frontotemporal dementia in the Genetic Frontotemporal dementia Initiative (GENFI) study: a cross-sectional analysis. Lancet Neurol. 14, 253–262. 10.1016/S1474-4422(14)70324-2 25662776 PMC6742501

[B29] Rohrer J. D. Lashley T. Schott J. M. Warren J. E. Mead S. Isaacs A. M.. (2011). Clinical and neuroanatomical signatures of tissue pathology in frontotemporal lobar degeneration. Brain 134(Pt 9), 2565–2581. 10.1093/brain/awr198 21908872 PMC3170537

[B30] Schiff W. Caviness J. A. Gibson J. J. (1962). Persistent fear responses in rhesus monkeys to the optical stimulus of “looming”. Science 136, 982–983. 10.1126/science.136.3520.982 14498362

[B31] Seeley W. W. Crawford R. K. Zhou J. Miller B. L. Greicius M. D. (2009). Neurodegenerative diseases target large-scale human brain networks. Neuron 62, 42–52. 10.1016/j.neuron.2009.03.024 19376066 PMC2691647

[B32] Seifritz E. Neuhoff J. G. Bilecen D. Scheffler K. Mustovic H. Schachinger H.. (2002). Neural processing of auditory looming in the human brain. Curr. Biol. 12, 2147–2151. 10.1016/S0960-9822(02)01356-8 12498691

[B33] Stecker G. C. Hafter E. R. (2000). An effect of temporal asymmetry on loudness. J. Acoust. Soc. Am. 107, 3358–3368. 10.1121/1.429407 10875381

[B34] Struhal W. Javor A. Brunner C. Benesch T. Schmidt V. Vosko M. R.. (2014). The phoenix from the ashes: cardiovascular autonomic dysfunction in behavioral variant of frontotemporal dementia. J. Alzheimers. Dis. 42, 1041–1046. 10.3233/JAD-140531 25024313

[B35] Sturm V. E. Rosen H. J. Allison S. Miller B. L. Levenson R. W. (2006). Self-conscious emotion deficits in frontotemporal lobar degeneration. Brain 129(Pt 9), 2508–2516. 10.1093/brain/awl145 16844714

[B36] Tyll S. Bonath B. Schoenfeld M. A. Heinze H. J. Ohl F. W. Noesselt T. (2013). Neural basis of multisensory looming signals. Neuroimage 65, 13–22. 10.1016/j.neuroimage.2012.09.056 23032489

[B37] Wang C. A. Boehnke S. E. White B. J. Munoz D. P. (2012). Microstimulation of the monkey superior colliculus induces pupil dilation without evoking saccades. J. Neurosci. 32, 3629–3636. 10.1523/JNEUROSCI.5512-11.2012 22423086 PMC6703448

[B38] Wang C. A. Munoz D. P. (2014). Modulation of stimulus contrast on the human pupil orienting response. Eur. J. Neurosci. 40, 2822–2832. 10.1111/ejn.12641 24911340

[B39] Warren J. D. Fletcher P. D. Golden H. L. (2012). The paradox of syndromic diversity in Alzheimer disease. Nat. Rev. Neurol. 8, 451–464. 10.1038/nrneurol.2012.135 22801974

[B40] Warren J. D. Rohrer J. D. Rossor M. N. (2013a). Clinical review. Frontotemporal dementia. BMJ 347:f4827. 10.1136/bmj.f4827 23920254 PMC3735339

[B41] Warren J. D. Rohrer J. D. Schott J. M. Fox N. C. Hardy J. Rossor M. N. (2013b). Molecular nexopathies: a new paradigm of neurodegenerative disease. Trends Neurosci. 36, 561–569. 10.1016/j.tins.2013.06.007 23876425 PMC3794159

[B42] Zhou J. Seeley W. W. (2014). Network dysfunction in Alzheimer's disease and frontotemporal dementia: implications for psychiatry. Biol. Psychiatry 75, 565–573. 10.1016/j.biopsych.2014.01.020 24629669

