# Supplementary material for: Dementias show differential physiological responses to salient sounds
Source: Front Behav Neurosci. 2015 Mar 24;9:73. doi: 10.3389/fnbeh.2015.00073 (PMC4373266; doi:10.3389/fnbeh.2015.00073)
Supplement: Supplementary file 1 [file Table1.DOCX]

**SUPPLEMENTARY MATERIAL**

**Dementias show differential physiological responses to salient sounds,** by PD Fletcher et al

| **‘different’ sound pairs** | |
| --- | --- |
|  |  |
| baby coughing | man snoring |
| baby laugh | waves lapping |
| baby screaming | man wheeze |
| baby sneezing | engine running |
| brushing teeth | train horn |
| car alarm being disarmed | shovel on metal |
| car horns | grandfather clock |
| car skidding | man snoring |
| cat whining | puppy yelping |
| child yawning | geese honking |
| clapping hands | dog barking |
| crashing waves | woman giggling |
| cuckoo clock | man breaking wind |
| dog growling | female cough |
| dog lapping | hiccupping |
| fizzy drink can being opened | coin being dropped on a table |
| crying | car crash |
| phone being hung up | female cough |
| hen clucking | man sighing |
| horse hooves | man walking on gravel |
| horse whinny | woman yawning |
| infant vocalising | stream |
| man crying pain | car horn |
| man sobbing | bees |
| mosquito | woman screaming |
| paper rustling | woman clears throat |
| paper tearing | hiccupping |
| phone dialling tone | man clears throat |
| phone engaged tone | cat hissing |
| phone ringing | man breathing |
| small bird cooing | man clicking fingers |
| small bird flapping wings | man eating |
| shovel digging in gravel | car window winding up |
| small bird flapping wings | graveyard Wind |
| thunder | car engine starting |
| clock ticking | baby crying |
| train on tracks | baby cough |
| vomiting | alarm bell |
| woman humming | trickling water |
| **‘same’ sound pairs** | |
|  |  |
| baby screaming | baby crying |
| car crashing | car horn |
| car horns | car skidding |
| child yawning | child hiccupping |
| cockerel | chicken |
| cuckoo clock | grandfather clock |
| geese honking | large wings flapping |
| horse whinny | horse hooves |
| infant vocalising | baby laughing |
| man clearing throat | man sighing |
| man crying in pain | vomiting |
| man sobbing | man breaking wind |
| phone ringing | dialling tone |
| pigeon wings flapping | pigeon cooing |
| stream | waves lapping on a shore |
| clock ticking | alarm bell |
| train on tracks | train horn |
| trickling water | crashing waves |
| woman clearing throat | woman yawning |
| woman crying | woman screaming |
| woman giggling | woman humming |

**Table S1.** Sound pairs used in the semantic classification task: Sounds in each pair were presented at fixed mean intensity and serially with a 1 second inter-sound gap; sound pairs were presented in randomised order. The task on each trial was to decide if the source of each sound was the same or different (‘Are the sounds made by the same kind of thing or different kinds of things?’)

| **Sound** | **Controls** | | **SD** | **PNFA** | **AD** | **sp-bvFTD** | **C9orf72** | **MAPT** | |
| --- | --- | --- | --- | --- | --- | --- | --- | --- | --- |
| ***Alerting ratings*** | | | | | | | | | |
| Iup | 7.42 (0.12)**^a^** | | 7.99 (0.34) | 6.32 (0.49) | 7.63 (0.24) | 7.83 (0.59) | 7.15 (0.23) | 7.61 (0.50) | |
| Idown | 7.11 (0.13) | | 7.29 (0.47) | 6.66 (0.46) | 7.75 (0.21) | 7.94 (0.45) | 7.12 (0.35) | 6.87 (0.54) | |
| ***Pupilmax*** | | | | | | | | | |
| Iup | | 0.17 (0.01)**^b^** | 0.13 (0.01)**^b^** | 0.13 (0.01)**^c^** | 0.15 (0.01)**^d^** | 0.18 (0.02) | 0.09 (0.02)**^e^** | | 0.16 (0.02) |
| Idown | | 0.14 (0.01) | 0.10 (0.02) | 0.15 (0.01) | 0.16 (0.02) | 0.16 (0.02) | 0.07 (0.03) | | 0.14 (0.02) |

**Table S2.** Summary of behavioural rating and pupil response data for participant groups: Mean alerting ratings and maximal pupil responses for the experimental groups for approaching (intensity increasing, **Iup**) and withdrawing (intensity decreasing, **Idown**) sound conditions. Alerting ratings are on a Likert scale (1, not all alerting; 10, highly alerting) and pupil responses are shown as log percentage maximal area change from baseline (Pupilmax). Mean values (standard errors) are shown. Key: **a**, strong trend (p<0.07) to greater alerting ratings for **Iup** than **Idown** sounds; **b**, significantly greater (p<0.05) pupil responses to **Iup** than **Idown** sounds; **c,** differential response to **Iup** vs **Idown** sounds significantly reduced (p<0.05) relative to healthy control and SD groups; **d,** differential response to **Iup** vs **Idown** sounds significantly reduced (p<0.05) relative to healthy control group; **e**, overall pupil responses significantly reduced (p<0.05) relative to healthy older controls and sporadic bvFTD subgroup; AD, Alzheimer’s disease; C9orf72, behavioural variant frontotemporal dementia with C9orf72 gene mutations; control, healthy older control group; MAPT, behavioural variant frontotemporal dementia with MAPT gene mutations; PNFA, progressive non-fluent aphasia; SD, semantic dementia; sp-bvFTD, sporadic behavioural variant frontotemporal dementia (no identified genetic mutations).
